# New evidence for T-cadherin in COVID-19 pathogenesis, endothelial dysfunction, and lung fibrosis

**DOI:** 10.3389/fcell.2025.1476329

**Published:** 2025-03-05

**Authors:** Ekaterina Semina, Vladimir Popov, Nikita Khabibullin, Polina Klimovich, Veronika Sysoeva, Ella Kurilina, Zoya Tsokolaeva, Vsevolod Tkachuk, Kseniya Rubina

**Affiliations:** ^1^ Faculty of Medicine, Lomonosov Moscow State University, Moscow, Russia; ^2^ Institute of Experimental Cardiology, National Cardiology Research Center of the Ministry of Health of the Russian Federation, Moscow, Russia

**Keywords:** *CDH13*, T-cadherin, COVID-19, endothelial dysfunction, lung fibrosis, angiotensin II

## Abstract

The COVID-19 pandemic had an unprecedented impact on all aspects of human activity worldwide, frequently resulting in post-acute sequelae and affecting multiple organ systems. The underlying mechanisms driving both acute and post-acute manifestations of COVID-19 are still poorly understood, warranting further investigation for new targets. The study represents the first attempt to explore the role of T-cadherin in COVID-19 pathogenesis as well as its implications in pulmonary fibrosis and endothelial dysfunction. First, we revealed a significant decrease in T-cadherin expression in post-mortem lung samples from COVID-19 patients. This downregulated T-cadherin expression correlated with the elevated levels of VE-cadherin and reduced levels of β-catenin, suggesting a disruption in endothelial cell-cell contact integrity and function. Second, the reciprocal relation of T-cadherin and VE-cadherin expression was further confirmed using cultured human endothelial Ea.hy926 cells. T-cadherin overexpression caused a decrease in VE-cadherin mRNA expression in cultured endothelial cells providing additional evidence in favor of their interplay. Third, employing *Cdh13* ^−/−^ mice, we unveiled the protective role of T-cadherin deficiency against bleomycin-induced lung fibrosis. Fourth, we demonstrated the mice lacking T-cadherin to have downregulated reactive oxygen species production and Nox2 mRNA expression in an angiotensin II-mediated endothelial dysfunction model. Our findings provide rationale for further studies into T-cadherin-mediated mechanisms in these processes.

## 1 Introduction

The pandemic of COVID-19, triggered by coronavirus 2 (SARS-CoV-2), had an enormous impact on the global healthcare. It is well-known that SARS-CoV-2 infection primarily targets the respiratory system, but there is evidence that it significantly affects the vasculature causing endothelial dysfunction and multiple organ injury (Xu et al., 2023; Zheng et al., 2023). Dysregulated immune response contributes to the ongoing inflammation and tissue damage in COVID-19. Hypercytokinemia is initiated by excessive production of proinflammatory mediators such as IL-1B, IL-6, IL-7, IL-8, IL-9, IL-10, IL-17, TNF-α among other cytokines (Caterino et al., 2021; Scherer et al., 2022; Gonzalez-Garcia et al., 2023). Adiponectin, a key adipokine produced by adipose tissue, is associated with regulation of lipid metabolism and inflammatory factor secretion in patients with SARS-CoV-2 (Grewal and Buechler, 2023; Kinoshita et al., 2023; Perrotta et al., 2023). AdipoR1, AdipoR2, and T-cadherin are the main receptors for adiponectin, and the ligand binding to these receptors activates a range of signaling pathways (Rubina et al., 2021). Although T-cadherin was initially described as an adhesion molecule, its functional repertoire goes beyond the limits of homophilic adhesion to operating as a receptor for low density lipoproteins (LDL) and high molecular weight (HMW) adiponectin (Rubina et al., 2021).

T-cadherin belongs to the cadherin superfamily, which comprises cell adhesion molecules intricately involved in a wide array of biological processes. Cadherins are crucial for embryogenesis and morphogenesis, angiogenesis, neurogenesis, or even neurotransmission. Cadherins act as membrane signaling receptors, triggering the activation of small GTPases and pathways involving β-catenin/Wnt, as well as regulating cytoskeletal polymerization at adhesive contact points. T-cadherin stands out as a distinctive member among cadherins: it possesses five extracellular Ca^2+^-binding domains typical of “classical” cadherins, yet lacks the transmembrane and cytoplasmic parts and is tethered to the plasma membrane via a glycosylphosphatidylinositol (GPI) anchor (Ranscht and Dours-Zimmermann, 1991). This GPI-anchor in the structure of T-cadherin enables its rapid movements within the plasma membrane and lateral interactions with numerous signaling partners (Rubina et al., 2021).

T-cadherin is highly expressed in human vasculature, particularly in large vessels (aorta, carotid, iliac and renal arteries), including endothelial cells, smooth muscle cells, and pericytes (Ivanov et al., 2001). Elevated T-cadherin expression in blood vessels correlates with progression of atherosclerosis, post-angioplasty restenosis, and tumor neovascularization (Ivanov et al., 2001; Kudrjashova et al., 2002; Adachi et al., 2006). Using *in vitro* models, T-cadherin was shown to regulate endothelial cell polarization and adhesion via homophilic interaction and subsequent activation of RhoA-ROCK- and Rac-dependent signaling (Philippova et al., 2005; Semina et al., 2014). Moreover, T-cadherin overexpression in endothelial cells promoted cell proliferation, migration and survival via activating PI3K/mTOR signaling pathway (Philippova et al., 2008; Philippova et al., 2012). In contrast, our *ex vivo* experiments demonstrated that T-cadherin overexpression in stromal cells suppressed neovascularization of the Matrigel plug by impeding the endothelial cell migration, capillary growth, and tube formation (Rubina et al., 2007). Furthermore, T-cadherin was shown to regulate endothelial permeability via clathrin-dependent endocytosis of VE-cadherin from the plasma membrane. This process led to the degradation of VE-cadherin in lysosomes, ultimately resulting in an increased endothelial permeability (Semina et al., 2014). In line with this, Wang and colleagues provided evidence suggesting that T-cadherin deficiency was associated with endothelial dysfunction (Wang et al., 2017). Using aortic rings from T-cadherin knockout mice, they demonstrated a significant impairment in acetylcholine-induced vasodilation compared to the control animals. This dysfunction was linked to decreased levels of NO_x_ and phospho-Akt (Wang et al., 2017). Therefore, T-cadherin expression and activity is intricately intertwined with intracellular signaling, angiogenesis and endothelial barrier function, and once impaired, T-cadherin expression correlates with endothelial dysfunction.

Besides homophilic interaction, T-cadherin’s functions as a receptor for HMW adiponectin (Hug et al., 2004). Adiponectin exerts multiple protective effects, including insulin-sensitizing, anti-inflammation, anti-proliferation, and anti-atherosclerotic actions (Achari and Jain, 2017). Specifically, T-cadherin binding with HMW adiponectin, the most metabolically active form of adiponectin, ensures its cardioprotective effects (Fujishima et al., 2017), enabling revascularization of the damaged skeletal muscles and myocardial recovery after injury (Denzel et al., 2010; Parker-Duffen et al., 2013). Towards that end, the decrease in plasma adiponectin levels is a hallmark of atherosclerosis, dyslipidemia, metabolic disorders and cardiovascular disease [cited in (Rubina et al., 2021)]. Consistent with this, it has been found that decreased expression of T-cadherin impairs adiponectin binding to endothelium and abrogates its protective effects (Parker-Duffen et al., 2013; Matsuda et al., 2015).

Systemic adiponectin level positively correlates with proper lung function in healthy adults. Reduced circulating adiponectin levels have been associated with severe subclinical lung inflammation, fibrosis, and diminished lung function [source (Kim et al., 2020)]. Exogenous administration of adiponectin was shown to reduce the allergic airway responses in mice including airway hyperresponsiveness and inflammation (Williams et al., 2012).

In the light of COVID-19 studies, adiponectin levels were found to be mostly lower, especially in respiratory failure, pointing to associations of adiponectin with the severity of COVID-19 (Barbalho et al., 2023; Grewal and Buechler, 2023). Serum adiponectin levels and the expression of adiponectin receptors (AdipoR1, AdipoR2, and T-cadherin) are closely related (Matsuda et al., 2015; Nguyen, 2020). Therefore, the adiponectin-T-cadherin system represents a promising target for investigating COVID-19 pathogenesis. While the role of adiponectin has been addressed in several studies (Barbalho et al., 2023), the contribution of T-cadherin remains largely unexplored. In the study by Lester J. Rosario-Rodríguez et al., it was shown that a decrease in the plasma level of T-cadherin correlates with the severity of the disease course in COVID-19 (Rosario-Rodríguez et al., 2024).

To investigate the potential role of T-cadherin in endothelial dysfunction and lung fibrosis with the underlying cellular and molecular mechanisms, we conducted a comprehensive analysis employing the following approaches: (1) Immunohistochemical staining to examine T-cadherin and the expression of related endothelial markers using autopsy material from the lungs of healthy donors and COVID-19 patients; (2) *In vivo* model of bleomycin-induced lung fibrosis using wild-type C57BL/6J mice and T-cadherin knockout (*Cdh13* ^−/−^) mice; (3) *In vivo* model of endothelial dysfunction induced by a 10-week administration of angiotensin II in wild-type C57/Bl mice and T-cadherin knockout (*Cdh13* ^−/−^) mice; (4) *In vitro* model of cultured endothelial cells with varying levels of T-cadherin expression to assess the target gene expression using qPCR.

## 2 Materials and methods

### 2.1 Human lung tissue samples

Post-mortem lung tissue samples (n = 10) were obtained from unvaccinated patients with SARS-CoV-2 infection as confirmed by PCR. Control lung tissue samples (n = 10) were collected from healthy donors, victims of car accidents. Patient samples were acquired in the National Medical Research Center of Cardiology named after Academician E.I. Chazov (Moscow, Russia) between April and July 2021. Demographic, clinical, and laboratory data were recorded upon patient admission. The study protocol was approved by and conducted according to the requirements of the ethics committee at the National Medical Research Center of Cardiology, named after Academician E.I. Chazov (Moscow, Russia), Protocol No. 249, 30 September 2019. According to article 68 of Federal Law of the Russian Federation of 21 November 2011 No. 323-FZ “About bases of protection of public health in the Russian Federation”, obtaining the consent from relatives/guardians is not required when analyzing human autopsy material and postmortem samples from unclaimed bodies. All patient data were completely anonymized.

### 2.2 Immunohistochemistry, microscopy, and morphometric analysis of human lung tissues

Human lung parenchymal tissues were fixed in formalin (Sintacon, Russia), embedded in paraffin (BioVitrum, Russia), cut into 5 μm sections further mounted on glass slides. Deparaffinization was performed using xylene (BioVitrum, Russia), followed by rehydration in sequentially decreasing concentrations of methanol (100%, 95%, and 70%) (Ecolan, Russia). Antigen unmasking was carried out using the Trilogy buffer (Sigma-Aldrich, Germany) according to the manufacturer’s protocol. The slides were washed twice with a PBS buffer (Dia-m, Russia) for 5 min each.

Endogenous peroxidase activity was blocked by incubation with 3% hydrogen peroxide (Samaramedprom, Russia) for 20 min, followed by two 5-min wash with PBS buffer (Dia-m, Russia). To prevent nonspecific binding, the samples were incubated with a 1% BSA/PBS solution (Dia-m, Russia) for 30 min at room temperature. Primary antibodies anti-T-cadherin (Affinity Biosciences, AF5203, USA), anti-VE-cadherin (Abcam, ab33168, USA), anti-β-catenin (Abcam, ab22656, USA) and anti-VCAM1 (Affinity Biosciences, DF6082, USA), anti-a-SMA (Dako, clone 1A4, USA), anti-CD31 (Dako, clone JC70A, USA), anti-E-cadherin (Santa Cruz, sc-8426, USA) were diluted to 1:100 in the blocking solution, applied to the slides and incubated for 1 h at room temperature. Then, the slides were incubated with Real EnVision peroxidase-conjugated secondary antibodies (Dako, USA) for 30 min at room temperature for single staining or with horse anti-mouse IgG AP antibodies, and ImmPRESS HRP/AP Polymer System (Vector Laboratories, USA) for 30 min at room temperature for double staining. Following incubation with secondary antibodies, the slides were washed three times with PBS for 5 min. For single staining, HiDef Detection™ HRP polymer detector (Cell Marque, USA) was applied for 10 min at room temperature, followed by a 5-min wash with PBS. Next, the chromogenic substrate DAB (DAKO, USA) was added for 2 min, and the reaction was terminated with distilled water. For double staining, ImmPACT DAB EqV substrate was applied, followed by ImmPACT Vector Red substrate (ImmPRESS HRP/AP polymer system, Vector Laboratories, USA) for 2 min. The reaction was stopped by adding of distilled water. The slides were dehydrated in the increasing concentrations of methanol (70%, 95%, 100%) and xylene. Finally, the samples were mounted in Cytoseal™ 60 permanent media (Thermo Scientific Richard - Allan Scientific).

Slides were scanned using Aperio ImageScope (v12.4.3.5008, Leica Microsystems GmbH). Quality control of the scanned images and further analysis were performed using Aperio ImageScope. The images were analyzed using the Positive Pixel Count V9 algorithm, which counts pixels of a given color, intensity, and saturation. The input parameters of the algorithm were initially set to identify pixels in brown or pink colors (positive pixels) and distinguish them from pixels of other colors (negative pixels). The results comprised quantitative values of positive (Np) and negative pixels (Nn); the final value of the marker expression level was determined using the formula: Np/(Np + Nn). Vessels were categorized as large vessels if their total cross-sectional area exceeded 5,000 μm^2^, and as small vessels if their total cross-sectional area was below 5,000 μm^2^. For analysis, one section was taken from each patient, and five measurements were performed per section.

### 2.3 Animals

All mice were sourced from in-house breeding program. Adult C57BL/6J T-cadherin knockout mice (*Cdh13* ^−/−^) were generated in our lab as described previously (Popov et al., 2023). *Cdh13* ^−/−^ and wild-type (WT) C57BL/6J mice were maintained in standard polypropylene cages under controlled vivarium conditions (temperature: 20°C–24°C, humidity: 35%–65%, 12-h light/dark cycle) with *ad libitum* access to food and water. Animal care and handling adhered to the European Convention for the Protection of Vertebrate Animals used for Experimental and other Scientific Purposes (ETS №123). A total of 15 WT mice and 15 *Cdh13* ^−/−^ mice (sex-matched), 6–14 weeks old, were enrolled into the study to provide statistical power. Mice were randomly assigned into two groups for subsequent experiments. Animal manipulations received ethical approval from the local ethical committee in accordance with the in-house requirements of the Commission on Bioethics of Lomonosov Moscow State University (license number 3.4). All procedures complied with Directive 2010/63/EU of the European Parliament and the Council of 22 September 2010 on the protection of animals used for scientific purposes.

### 2.4 Bleomycin-induced pulmonary fibrosis in mice

To model pulmonary fibrosis in mice, we utilized bleomycin-induced lung injury as described previously (Shmakova et al., 2023). *Cdh13* ^−/−^ (n = 11) and WT (n = 9) mice were intratracheally administered with either bleomycin (Nippon Kayaku, Tokyo, Japan, 3 mg/kg, 10 mg/mL solution) or saline (NaCl) (Bionit, Russia) as a control. Bleomycin administration was performed under isoflurane anesthesia using an air mixture containing 2%–4% isoflurane (Laboratorios Karizoo, S.A., Barcelona, Spain) and 93% oxygen (V3000 vaporizer, Parkland Scientific, Coral Springs, FL, USA, with Nuvo Lite 525 oxygen concentrator, Nidek medical products, Birmingham, AL, USA). Day 0 was defined as the day of bleomycin administration. Mice were monitored until day 14, when magnetic resonance imaging (MRI) analysis was conducted.

### 2.5 MRI for assessing lung fibrosis

Lung MRI was conducted using a ClinScan 7T tomograph (Bruker Biospin, Billerica, MA, USA) following the previously established protocols (Shmakova et al., 2023). Mice were anesthetized with an isoflurane-oxygen mixture (2%–4% isoflurane, 93% oxygen); respiration was monitored using an MR-compatible Model 1,025 Small Animal Monitoring and Gating System (Small Animal Instruments, Inc., Brookhaven, NY, USA). Fat-suppressed T2-weighted turbo-spin-echo sequences were employed to acquire lung MRI data with the following parameters: TR = 1,175 ms, TE = 55 ms, echo train length = 8, FOV 42 × 60 mm, base resolution 216 × 384.

For lung fibrosis assessment, the MRI frontal projection was utilized. Image analysis was performed using ImageJ software [National Institutes of Health, Bethesda, MA, USA) as described earlier (ImageJ, 2024)].

### 2.6 Model of endothelial dysfunction in mice

Endothelial dysfunction was induced using a modified version of the method by Trejo-Moreno et al. (2021). *Cdh13* ^−/−^ (n = 4) and WT (n = 4) male mice were enrolled. We utilized groups of n = 4 mice due to the complexity of histological analysis and qPCR, supported by previous studies demonstrating that 4 animals per group are sufficient to detect differences in NADPH oxidase expression in this model (Trejo-Moreno et al., 2021). In line with the Reduction principle, we aimed to minimize the number of animals used. Statistical power analysis, conducted with G-power software and based on NADPH oxidase expression data from Trejo-Moreno et al. (2021), yielded a power value of 0.9988 for a sample size of 4 animals, confirming its adequacy.

To mimic endothelial dysfunction, angiotensin II (human, vasoconstrictor peptide, Abcam, USA, cat. # ab120183) was administered intraperitoneally at a dose of 0.1 μg/kg (solution in sterile NaCl, 0.05 μg/mL) daily for 10 weeks. Systolic and diastolic blood pressure and heart rate measurements were performed in all mice on the same day, between 14:00 and 17:00 h (light phase), to minimize the effects of daily fluctuations. Prior to blood pressure measurements, mice were anesthetized with isoflurane (a mixture containing 1.5%–2% isoflurane (Laboratorios Karizoo, S.A., Barcelona, Spain) and approximately 93% oxygen) provided at a flow rate of 0.2–0.4 L/min using a V3000 Parkland Scientific vaporizer and a Nuvo Lite 525 oxygen concentrator. Anesthetized animals were placed on a heated mat (36°C–37°C) during measurements. Blood pressure measurements were performed using a digital plethysmograph “Sistola” (Neurobotics LLC, Russia). In our study’s endothelial dysfunction model, significant changes in both systolic and diastolic pressure were detected as early as the fifth week of angiotensin II injections (Trejo-Moreno et al., 2021). Therefore, blood pressure measurements were discontinued after the seventh week, when pronounced systolic pressure alterations became evident. However, angiotensin II administration was continued to enable further analysis of histological changes and qPCR in tissue samples. Baseline measurements were recorded on the first day of angiotensin II injections, followed by weekly measurements until day 49. On concluding the experiment, the mice were euthanized. The lungs and kidneys were then isolated after perfusion. Kidneys and lungs were weighed, snap-frozen in liquid nitrogen, and stored at −80°C until further processing.

### 2.7 Histological assessment of fibrosis development in kidneys and lungs and morphometric analysis of stained sections

To evaluate fibrosis or collagen deposition, sections (5 μm) of lung and kidney tissues were prepared. Histological staining was conducted using hematoxylin and eosin (H&E), picrosirius red, or Van Gieson dyes. Aperio ImageScope or Leica DM6000B (Leica Microsystems GmbH) was used to for tissue sections staining.

ImageJ software was used to quantify the area of collagen (determined by picrosirius red staining) or elastic fibers (determined by Van Gieson staining) staining in tissue sections. The color in the images was deconvoluted into three sublayers using the Image > Color > Color Deconvolution > H AEC function. The total area of the tissue section and the area occupied by the positively stained collagen or elastic fibers (bright colors) were determined on the selected layer using the Image > Adjustment > Threshold function. The extent of collagen or elastic fiber depositions were assessed by calculating the ratio of positively stained area relative to the total area of the sample (staining score). All images were analyzed excluding the internal lumen of blood vessels, and nonspecific staining in this region was eliminated using the Negative Pen tool. For analysis, one section was taken from each patient, and five measurements were performed per section.

The relative thickness of vessel walls was quantified as the percentage of vessel wall cross-sectional area (the difference between total vessel cross-sectional area and luminal cross-sectional area) relative to the total vessel cross-sectional area. The relative thickness of perivascular connective tissue was determined by measuring the area of connective tissue surrounding a blood vessel to the total area of the vessel and perivascular connective tissue. This ratio was expressed as a percentage.

### 2.8 Assessment of reactive oxygen species (ROS) production

The generation of ROS in mouse kidney and lung tissues was evaluated using the dihydroethidium (DHE) oxidation assay. The kidneys and lungs were homogenized in ice-cold HEPES buffer (10 mM HEPES (Sigma-Aldrich, Germany), 0.1 mM phenyl-methyl-sulfonyl-fluoride (Sigma-Aldrich, Germany)) and centrifuged at 10,000 × g for 10 min at 4°C, then the supernatants were collected. The conversion of DHE to ethidium (*E*th) was employed as a measure of ROS production. Supernatant aliquots (10 µL) were incubated with 0.2 mM DHE (Sigma-Aldrich, Germany), salmon testis DNA (10 mg/mL) (Thermo Scientific, USA), and the substrate for xanthine oxidase (XO, xanthine (0.1 mM)) (Sigma-Aldrich, Germany). Eth-DNA fluorescence was measured at an excitation wavelength of 480 nm and an emission wavelength of 610 nm at 37°C for 30 min, using a multimode microplate reader (Thermo Scientific, USA). Background fluorescence was determined using a blank sample lacking any biological material, and this value was subtracted from the reading of each sample.

### 2.9 IL-17 quantification by enzyme-linked immunosorbent assay (ELISA)

The mouse kidneys and lungs were homogenized in a pre-cooled mortar with ice-cold HEPES-PMSF (0.1%) buffer in a 1:7 w/v ratio. The homogenates were centrifuged at 10,000 × g for 15 min at 4°C, and the supernatants were collected and stored at −20°C until further analysis.

IL-17 protein levels were quantified using an ELISA kit (Cloud Clone, cat. # SEA063Mu, USA) following the manufacturer’s instructions. Briefly, lung and kidney extracts were added to a 48-well plate and incubated for 1 h at 37°C. The plates were incubated with the corresponding anti-IL-17-HRP detection antibodies for 30 min at 37°C. Bound immune complexes were visualized by reaction with tetramethylbenzidine (TMB) substrate after 30 min of incubation in the dark. The reaction was terminated by 1M H_2_SO_4_ (PanReac, Spain) added, and the absorbance was measured at 450 nm using a VERSAmax ELISA plate reader (Molecular Devices). IL-17 concentrations were determined based on a standard protein dilution curve and expressed as pg/mL protein.

### 2.10 Cell culture

Human endothelial cell line Ea.hy926 (ATCC CRL-2922™) was used in this study. The cells were seeded onto culture flasks at a density of 10^4^ cells/cm^2^ in a complete DMEM medium supplemented with 10% serum (HyClone, USA), and maintained in a CO_2_ incubator at 37°C.

For T-cadherin overexpression, we employed the pcDNA3.1-DsRed-Tcad plasmid encoding human *CDH13* cDNA, a kind gift from the Laboratory of Cellular Engineering led by T.N. Vlasik, at the Federal State Budgetary Institution National Medical Research Center of Cardiology of the Ministry of Health of the Russian Federation. As a control, we used the pcDNA3.1-DsRed plasmid. Cells were transfected using Lipofectamine 2000 (Invitrogen, USA). Both plasmids encoded the red fluorescent protein gene DsRed, enabling post-transfection selection using a BD FACSAria III cell sorter (BD Biosciences, USA). Following cell sorting, the selected cells were cultured and expanded for RNA isolation and gene expression analysis.

### 2.11 RNA extraction, reverse transcription and quantitative PCR

Total RNA from Ea.hy926 human endothelial cells was extracted using the NucleoSpin RNA kit (Macherey-Nagel, Germany) following the manufacturer’s protocol. Total RNA from mouse kidney and lung tissues was isolated using Extract RNA reagent (Evrogen, Russia) according to the manufacturer’s instructions. RNA quantity and quality were evaluated using a NanoDrop 1000 spectrophotometer (Thermo Fisher Scientific, USA).

One microgram of total RNA was reverse transcribed using oligo (dT) and random (dN)_10_ primers with the MMLV RT kit (Evrogen, Russia). Quantitative PCR (qPCR) was performed using qPCRmix-HS SYBR (Evrogen, Russia) on a CFX96 Touch real-time PCR instrument (BioRad, USA). Primers were designed with the Primer Blast designing tool and assessed for quality by OligoAnalyser (Table 1). Primers were synthesized by Evrogen (Russia). The thermal cycling program comprised a 5-min denaturation step at 95°C, followed by 40 amplification cycles of 15-s denaturation at 95°C, 15-s annealing at 62°C, and 20-s extension at 72°C. qPCR for each sample was performed in triplicate. Relative transcript levels were determined using the 2^−ΔΔCt^ method with *Rpl13a* or *RPLP0* as the reference genes. Normalization was performed by setting the average transcript level in control to 1. Outliers were identified and excluded using the regression method (ROUT).

**TABLE 1:** The sequences of murine and human primers used in the study for RT-qPCR.

Gene	Forward primer	Reverse primer
Murine primers		
*Nox2*	5′-AGG​AGT​GCC​CAG​TAC​CAA​AGT-3′	5′-TAC​TGT​CCC​ACC​TCC​ATC​TTG-3′
*Nox4*	5′-ACC​CAA​GTT​CCA​AGC​TCA​TTT-3′	5′-ATG​GTG​ACA​GGT​TTG​TTG​CTC-3′
*Nos2*	5′-CCA​AGC​CCT​CAC​CTA​CTT​CC-3′	5′-CTC​TGA​GGG​CTG​ACA​CAA​GG-3′
*Nos3*	5′-CAA​CGC​TAC​CAC​GAG​GAC​ATT-3′	5′-CTC​CTG​CAA​AGA​AAA​GCT​CTG​G-3′
*Icam1*	5′-GTG​ATG​CTC​AGG​TAT​CCA​TCC​A-3′	5′-CAC​AGT​TCT​CAA​AGC​ACA​GCG-3′
*Rpl13a*	5′-CCC​CAC​AAG​ACC​AAG​AGA​GG-3′	5′-CCC​CAG​GTA​AGC​AAA​CTT​TCT​G-3′
Human primers		
*CTNNB1*	5′-ACG​GAG​GAA​GGT​CTG​AGG​A-3′	5′-CCA​ACT​CCA​TCA​AAT​CAG​CTT​G-3′
*CDH5*	5′-AAG​CGT​GAG​TCG​CAA​GAA​TG-3′	5′-TCT​CCA​GGT​TTT​CGC​CAG​TG-3′
*CDH13*	5′-TAT​GGC​AGA​ACT​CGT​GAT​TGT​CG-3′	5′-CTG​TCA​CTA​TCG​ACT​ACC​TTG​C-3′
*RPLP0*	5′-TCG​AAC​ACC​TGC​TGG​ATG​AC-3′	5′-GCA​CCA​TTG​AAA​TCC​TGA​GTG​A-3′

### 2.12 Statistical analysis

Data analysis was performed using GraphPad Prism 9 software (GraphPad Software Inc.). The Mann-Whitney test was employed to compare the differences between two groups. Two-way ANOVA followed by the Holm-Šidák *post hoc* test was utilized to evaluate differences between two or more groups involving two factors. Heart rate data, along with changes in systolic and diastolic blood pressure, were analyzed using repeated measures ANOVA test. Data are presented as median [interquartile range]. A *p*-value less than 0.05 was considered statistically significant.

## 3 Results

### 3.1 COVID-19 dysregulated expression of endothelial adhesion molecules in human lungs

**FIGURE 1:**
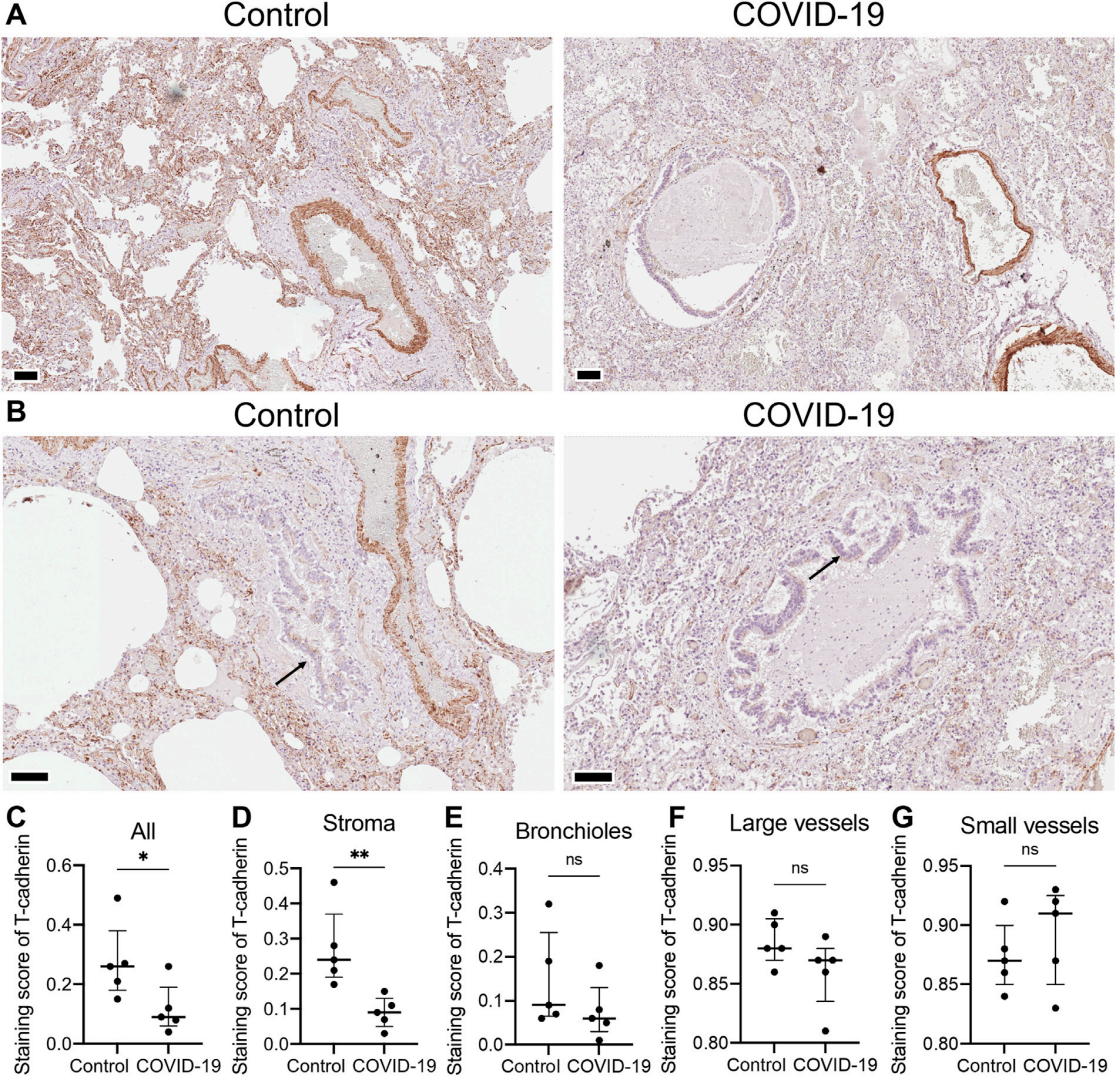
T-cadherin expression detected in lung tissues of healthy controls and COVID-19 patients. **(A)** Representative sections of parenchymal lung tissue from a healthy control and a COVID-19 patient stained for T-cadherin as revealed with DAB HRP Substrate (brown) and counterstained with hematoxylin. Scale bar 100 μm. **(B)** Representative sections of the hilar region of the lung tissue from a healthy control and a COVID-19 patient stained for T-cadherin as revealed with DAB HRP Substrate (brown). Arrows point to T-cadherin expression. Scale bar 100 μm. **(C–G)** T-cadherin staining scores in the images of lung tissue sections of COVID-19 patients and control healthy donors in the tissue as a whole, **(C)**, stroma **(D)**, bronchioles **(E)**, large **(F)** and small blood vessels **(G)**. Images were analyzed by the Positive Pixel Count V9 algorithm of ImageScope (Aperio), which counts pixels of the predetermined color (brown for T-cadherin, positive pixels) and pixels related to other colors (negative pixels). A staining score was calculated as the number of positive pixels/(number of positive + negative pixels). Data are shown as individual values, median and interquartile range, ns - non-significant, *p < 0.05, **p < 0.01, Mann-Whitney test.

To understand the differential expression of T-cadherin in healthy and diseased human lungs, we first assessed the presence of T-cadherin in the lung tissues of COVID-19 patients and healthy controls by immunohistochemistry (Figures 1A, B). T-cadherin was abundantly expressed in the stroma and blood vessels in healthy lungs (Figure 1A). A significant overall decrease in T-cadherin expression was found in lung tissues of COVID-19 patients compared to normal lungs (*p* = 0.0397, Mann-Whitney, Figure 1C). This decrease was detected in lung stroma (*p* = 0.0079, Mann-Whitney, Figure 1D), but not in bronchioles or blood vessels (Figures 1E–G). T-cadherin in bronchioles was expressed on the apical surface of the ciliated epithelium in both normal and COVID-19 bronchioles with some heterogeneity (Figure 1B). This suggests that the expression of T-cadherin in bronchioles may be highly variable and dependent on bronchiole size.

**FIGURE 2:**
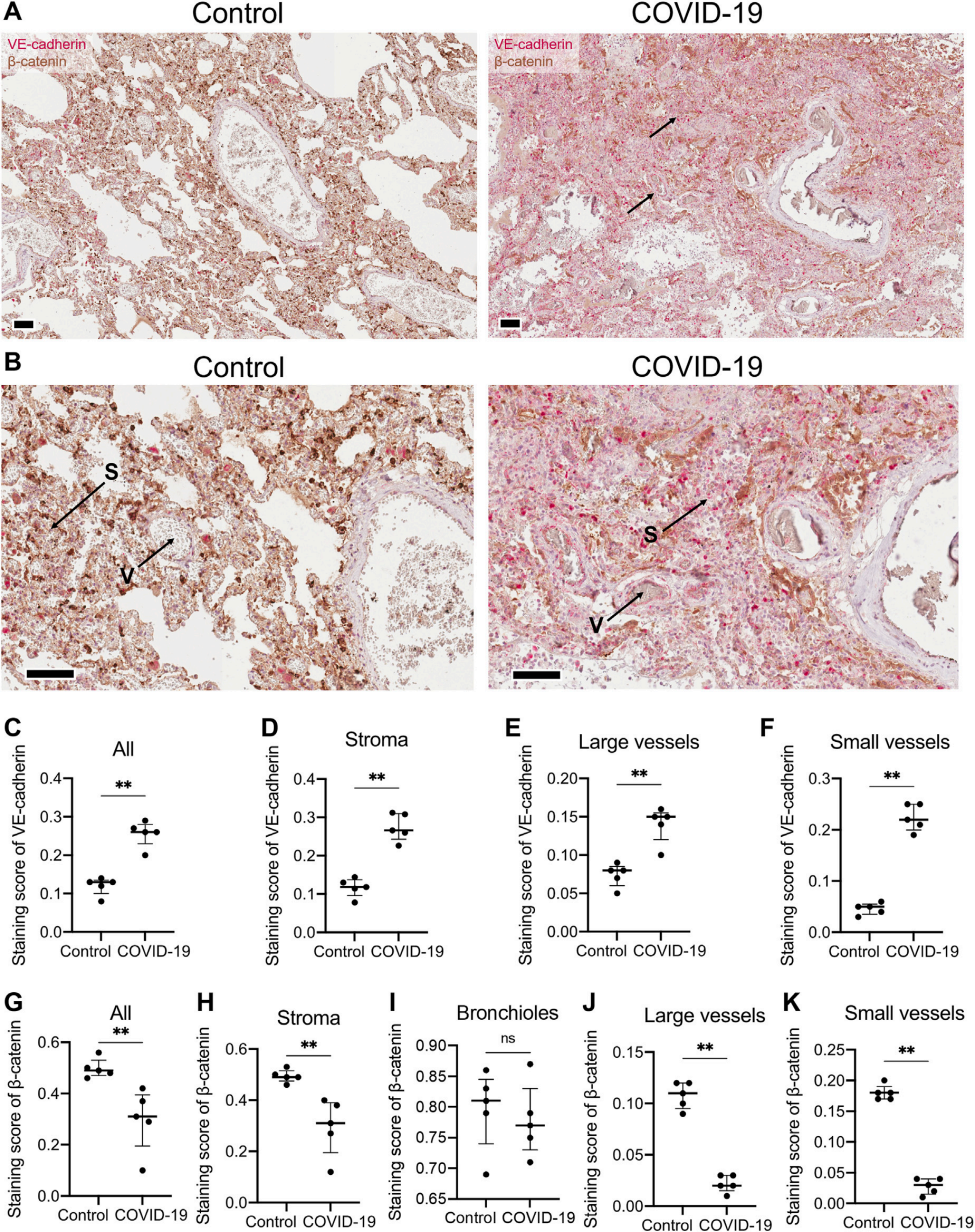
VE-cadherin and β-catenin expression detected in lung tissue of healthy controls and COVID-19 patients. **(A)** Representative section of parenchymal lung tissues from a healthy control and a COVID-19 patient stained for VE-cadherin (revealed with ImmPACT Vector Red Substrate, pink) and β-catenin (revealed with DAB HRP Substrate, brown), and counterstained with hematoxylin. Arrows point to VE-cadherin expression. Scale bar 100 μm. **(B)** Enlarged image of the illustrations from panel **(A)**. Vessels (V) and stroma (S) are indicated. **(C–F)** VE-cadherin staining scores in the images of lung tissue sections from COVID-19 patients and control healthy donors in the whole tissue **(C)**, stroma **(D)**, large **(E)** and small blood vessels **(F)**. Because of a significant signal overlap between VE-cadherin and β-catenin, accurately calculating the staining score of VE-cadherin in the bronchioles was not feasible. **(G–K)** β-catenin staining scores in the images of lung tissue sections of COVID-19 patients and control healthy donors in the whole tissue **(G)**, stroma **(H)**, bronchioles **(I)**, large **(J)** and small blood vessels **(K)**. Images were analyzed by the Positive Pixel Count V9 algorithm of ImageScope (Aperio), which counts pixels of the predetermined color (pink for VE-cadherin and brown for β-catenin, positive pixels) and pixels related to other colors (negative pixels). A staining score was calculated as the number of positive pixels/(number of positive + negative pixels). Data are shown as individual values, median and interquartile range, ns - non-significant, **p < 0.01, Mann-Whitney test.

In endothelial cells, T-cadherin is involved in degrading vascular endothelial (VE)-cadherin, a key endothelial adhesion molecule that maintains endothelial cell integrity and promotes vascular stability (Semina et al., 2014). To further elucidate the impact of COVID-19 on vascular endothelial function, we examined the expression of VE-cadherin in lung sections of COVID-19 patients and healthy controls (Figures 2A,B). Immunohistochemical staining of lung tissues from COVID-19 patients revealed a significant upregulation of VE-cadherin expression compared to healthy individuals (*p* = 0.0079, Mann-Whitney, Figure 2C). This enhanced VE-cadherin immunostaining was evident in both the stroma (*p* = 0.0079, Mann-Whitney, Figure 2D) and in large (*p* = 0.0079, Mann-Whitney, Figure 2E) and small blood vessels (*p* = 0.0079, Mann-Whitney, Figure 2F).

To validate the observed reduction of T-cadherin expression in the lungs during COVID-19, we conducted double immunohistochemical (IHC) and double immunofluorescent staining on lung tissues using antibodies against T-cadherin and CD31 (vessels), E-cadherin (bronchioles), and the stromal marker α-SMA (Supplementary Figure S1 for double IHC; Supplementary Figure S2 for double immunofluorescent staining). However, due to nonspecific staining these data were excluded from further analysis.

We next examined the expression of β-catenin, a key mediator of the Wnt signaling pathway and intracellular partner of VE-cadherin (Nan et al., 2023). Immunohistochemical staining of lung tissue from COVID-19 patients revealed a significant downregulation of β-catenin compared to healthy individuals in the whole lung (*p* = 0.0079, Mann-Whitney, Figure 2G). The decline in β-catenin expression in COVID-19 lung samples was observed in lung stroma (*p* = 0.0079, Mann-Whitney, Figure 2H), large (*p* = 0.0079, Mann-Whitney, Figure 2J) and small blood vessels (*p* = 0.0079, Mann-Whitney, Figure 2K), but not in bronchioles (Figure 2I).

To further characterize the impact of COVID-19 on endothelial function, we examined the expression of vascular cell adhesion molecule-1 (VCAM-1), a crucial marker of endothelial activation that mediates the adhesion of leukocytes to vascular wall (Bermejo-Martin et al., 2020). Immunohistochemical staining of lung tissue from COVID-19 patients revealed an increase in VCAM-1 expression in small vessels compared to healthy donors (Supplementary Figure S3). Notably, under normal conditions, VCAM-1 expression was minimal in lung vessels, with the exception of nonspecific staining of erythrocytes in vascular lumen (Supplementary Figure S3). This observation is consistent with the recognized role of VCAM-1 as an endothelial activation marker, known to be upregulated during inflammatory responses, including COVID-19 infection (Xu et al., 2023). Interestingly, VCAM-1 expression was also detected in large vessels in both healthy and COVID-19 lung tissue (Supplementary Figure S3). This staining pattern reveals VCAM-1 expression in both the intima and media of large vessels, possibly indicating its presence in smooth muscle cells (SMCs). However, VCAM-1 is not typically expressed in vascular SMCs under normal conditions (Libby and Li, 1993) and may result from the specific method of lung sample collection from healthy donors, victims of car accidents. The trauma associated with these accidents could have elicited an inflammatory response in the lungs, resulting in the upregulation of VCAM-1 expression in SMCs.

### 3.2 T-cadherin overexpression in human endothelial cells downregulates VE-cadherin and β-catenin expression

To investigate further the potential association between T-cadherin, VE-cadherin, and β-catenin expression, we overexpressed T-cadherin in human endothelial cells. For T-cadherin overexpression, we transfected human endothelial cells Ea.hy926 with a pcDNA3.1-DsRed-Tcad plasmid encoding human *CDH13* cDNA (*CDH13* OE). As a control, cells were transfected with a pcDNA3.1-DsRed plasmid. The transfected cells were sorted based on DsRed expression, followed by RT-qPCR analysis.

**FIGURE 3:**
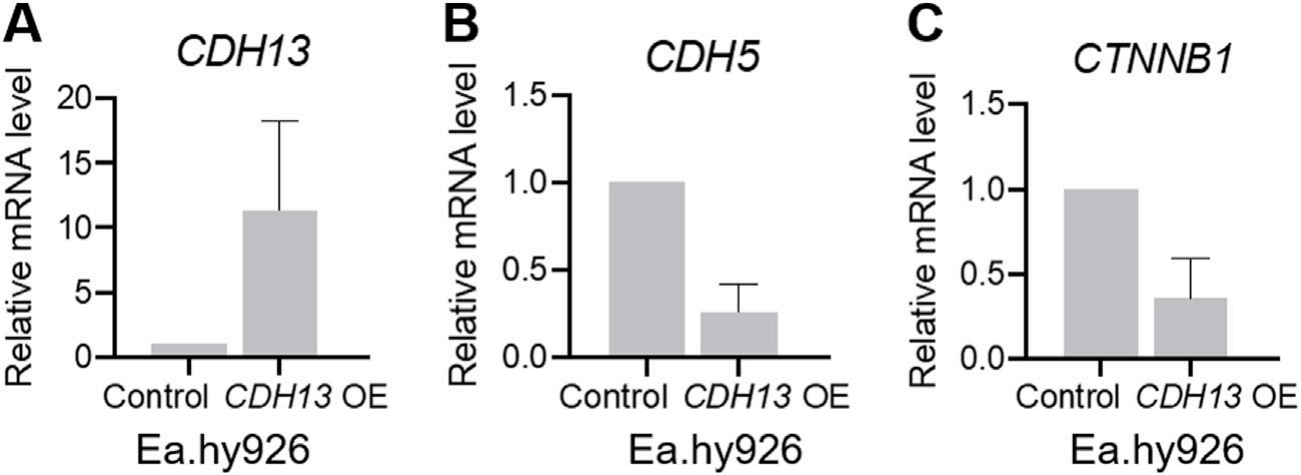
T-cadherin overexpression leads to dysregulation of gene expression in endothelial cells. Human endothelial cells Ea.hy926 were transfected with either a pcDNA3.1-DsRed plasmid (control) or a pcDNA3.1-DsRed-Tcad plasmid for T-cadherin overexpression (*CDH13* OE) and sorted based on DsRed expression, followed by RT-qPCR. **(A)** mRNA expression levels of *CDH13* in control and *CDH13* OE human endothelial Ea.hy926 cells. **(B, C)** mRNA expression levels of *CDH5* **(B)**, and *CTNNB1* **(C)** in control and *CDH13* OE human endothelial Ea.hy926 cells. Data are presented as median and interquartile range. Results from two biologically independent experiments, each performed in triplicate for technical reproducibility, are presented.

We confirmed that T-cadherin expression in *CDH13* OE cells was increased compared to the control cells (Figure 3A). To investigate the role of T-cadherin in VE-cadherin downregulation, we analyzed *CDH5* gene (encoding VE-cadherin) expression. Indeed, two independent experiments demonstrated that Ea.hy926 endothelial cells with *CDH13* OE exhibited a lower level of *CDH5* expression (0.2626 [0.109–0.416], Figure 3B). Next, we examined the expression of the *CTNNB1* gene (encoding β-catenin). In two independent experiments, we found that Ea.hy926 endothelial cells with *CDH13* OE displayed a reduced level of *CTNNB1* expression (0.3578 [0.122–0.594], Figure 3C).

Comparing the findings from human lung tissue samples of COVID-19 patients and cultured human endothelial Ea.hy926 cells, we observed a correlation between the elevated T-cadherin expression (noted in control vs COVID-19 patients and in *CDH13* OE endothelial cells vs control cells) and reduced VE-cadherin in both scenarios (Figures 1–3). However, an intriguing contrast emerged in β-catenin expression. In human lung tissue samples, the increased T-cadherin expression (detected in control vs COVID-19 patients) correlated with the elevated β-catenin (Figures 1, 2). Conversely, in human endothelial cells *in vitro*, the increased T-cadherin expression was associated with the reduced β-catenin levels (Figure 3). This suggests that in these settings, there is many a factor besides T-cadherin affecting the overall expression and function of adhesion proteins.

Our immunohistochemistry analysis of lung tissues of COVID-19 patients revealed heightened levels of VE-cadherin in both lung stroma and blood vessels along with a reduced expression of β-catenin in COVID-19 patients relative to healthy controls (Figure 2). We have previously demonstrated that overexpression of T-cadherin in cultured endothelial cells (HUVECs) triggers clathrin-dependent endocytosis of VE-cadherin with its subsequent degradation in lysosomes, resulting in the disruption of endothelial barrier function and increased permeability (Semina et al., 2014). Consistent with these data, our present results on human endothelial Ea.hy926 cells demonstrated a decrease in mRNA expression levels of both VE-cadherin and β-catenin upon T-cadherin overexpression (Figure 3). Our data on the elevated VE-cadherin expression (Figures 2, 3) in lung samples of COVID-19 patients are in line with the reduced T-cadherin expression (Figure 1), however are in contrast with the recently published results on reduced VE-Cadherin expression during SARS-CoV-2 viral infection (Nader and Kerrigan, 2022; Xu et al., 2023).

### 3.3 T-cadherin knockout mitigates bleomycin-induced pulmonary fibrosis

**FIGURE 4:**
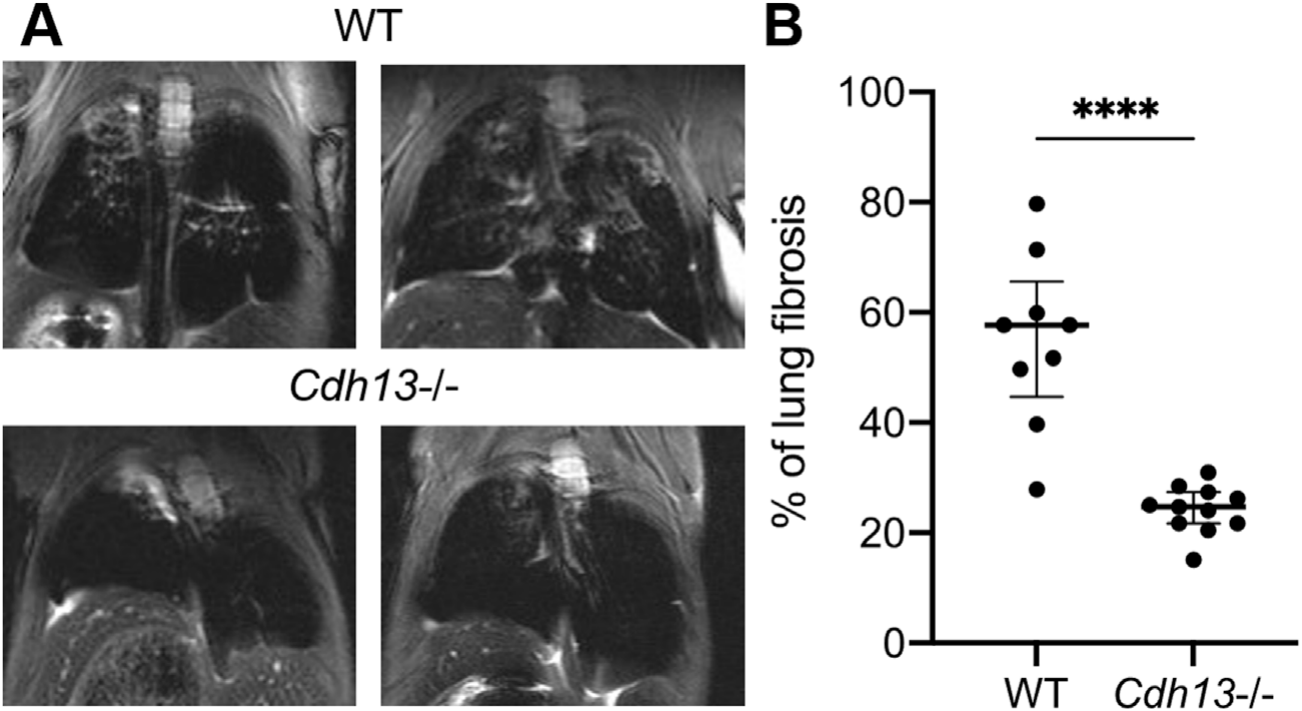
Differential progression of pulmonary fibrosis between wild-type (WT) and T-cadherin knockout (*Cdh13* ^*−/−*^) mice following bleomycin instillation. Bleomycin (3 mg/kg) was administered to both WT and *Cdh13* ^−/−^ mice by intratracheal instillation on day 0. **(A)** Typical MRI images obtained 28 days after bleomycin administration to *Cdh13* ^−/−^ and WT mice. The affected lung areas on the MRI images appear light, and the intact lungs appear dark. **(B)** The percentage of lung fibrotic tissue assessed on day 28 following bleomycin intratracheal instillation in WT and *Cdh13* ^−/−^ mice. Data are shown as individual values, median and interquartile range, ****p < 0.0001, Mann-Whitney test.

Our results presented above indicated that T-cadherin was downregulated in COVID-19 patients (Figure 1). To further evaluate the consequences of T-cadherin deficiency, we employed a bleomycin-induced lung injury model using WT and T-cadherin knockout (*Cdh13* ^−/−^) mice (Figure 4A). Bleomycin-induced lung injury closely resembles the pathological features of pulmonary fibrosis, a hallmark of severe COVID-19 (Shmakova et al., 2023). By day 28 post-bleomycin exposure, *Cdh13* ^−/−^ mice exhibited significantly reduced lung fibrosis compared to control WT mice (*p* < 0.0001, Mann-Whitney, Figure 4B). This observation implicates a protective mechanism of T-cadherin downregulation against the development of pulmonary fibrosis.

### 3.4 T-cadherin knockout increases vessel wall thickness upon angiotensin II-induced endothelial dysfunction

**FIGURE 5:**
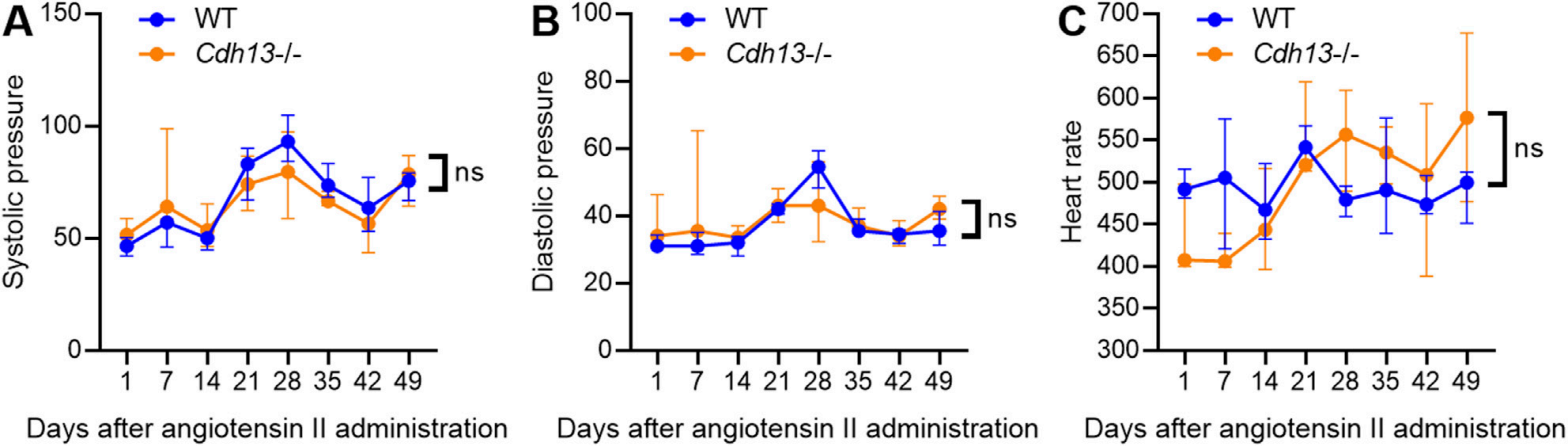
The dynamics of systolic blood pressure **(A)**, diastolic blood pressure **(B)** and heart rate **(C)** in WT and T-cadherin knockout (*Cdh13* ^−/−^) mice in response to angiotensin II. Data are presented as median and interquartile range, *n* = 4 in each group, ns - non-significant, Repeated Measures ANOVA test.

Since endothelial dysfunction is a risk factor for pulmonary fibrosis (Zhao et al., 2023) and T-cadherin downregulation in COVID-19 patients was associated with dysregulation of endothelial adhesion molecules (Figures 1, 2), we further explored the role of T-cadherin knockout (*Cdh13* ^−/−^) on angiotensin II-induced endothelial dysfunction in mice. Angiotensin II, a potent vasoconstrictor, was administered intraperitoneally daily to WT and *Cdh13* ^−/−^ mice for 10 weeks. Systolic and diastolic pressure was monitored continuously. We observed significant alterations in systolic pressure in both WT and *Cdh13* ^−/−^ mice throughout the study period (time factor *p* = 0.0010, two-way ANOVA, Figure 5A). However, no significant difference was found in systolic pressure between the two groups at any point, including the 49th day (Figure 5A). Additionally, no significant differences were detected in diastolic pressure over time or between the *Cdh13* ^−/−^ and WT mice groups (Figure 5B). Similarly, there were no significant changes in heart rate between *Cdh13* ^−/−^ vs. WT mice (Figure 5C).

**FIGURE 6:**
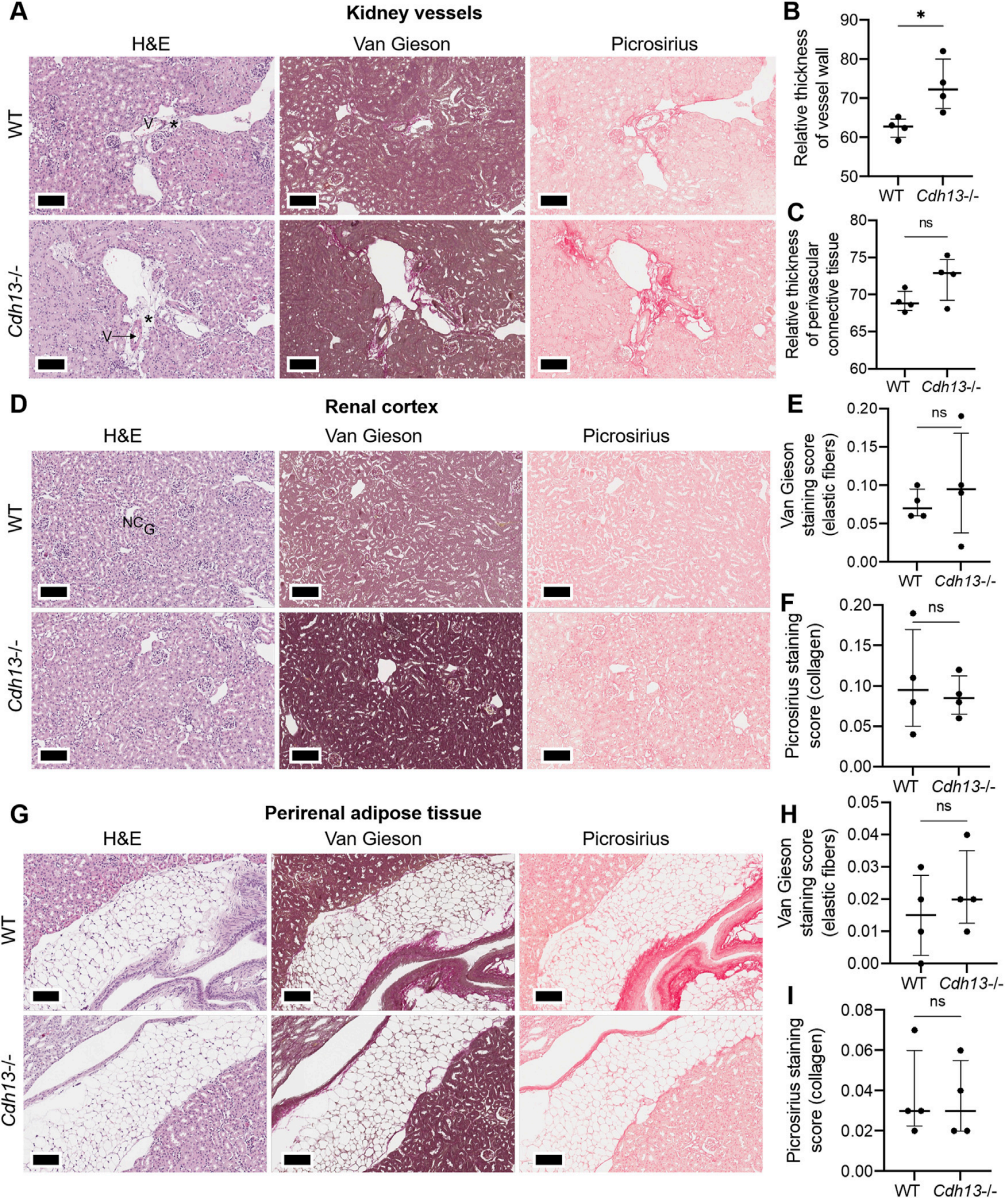
Histological assessment of fibrotic changes in the renal tissue of wild-type (WT) and T-cadherin knockout (*Cdh13* ^−/−^) mice on the 49th day following the angiotensin II administration. Tissue sections were stained with hematoxylin-eosin, Van Gieson, and picrosirius red. Vessels (V), nephron canal (NC), and glomerulus (G) are indicated, correspondingly. **(A)** Representative micrographs of histological staining of renal vessels. Asterisks point to the difference in vessel wall thickness. **(B)** Relative thickness of blood vessel walls is presented as the percentage of vessel wall area (the difference between the total vessel area and luminal area) relative to the total vessel area. **(C)** Relative thickness of perivascular connective tissue is presented as the percentage of connective tissue surrounding a blood vessel relative to the total area of the vessel and perivascular connective tissue. **(D)** Representative micrographs of histological staining of the renal cortex. **(E)** The Van Gieson staining score in the renal cortex presented as the ratio of positively stained area relative to the total area of the analyzed sample (staining score). **(F)** The picrosirius staining score in the renal cortex presented as the ratio of the positively stained area relative to the total area of the analyzed sample (staining score). **(G)** Representative micrographs of histological staining of the perirenal adipose tissue. **(H)** The elastic fibers picrosirius staining score in perirenal adipose tissue presented as the ratio of the positively stained area relative to the total area of the analyzed sample (staining score). **(I)** The collagen area picrosirius staining score in perirenal adipose tissue presented as the ratio of positively stained area relative to the total area of the analyzed sample (staining score). Scale bar 100 μm. Data are presented as individual values, median and interquartile range, ns - non-significant, *p < 0.05, Mann-Whitney test.

**FIGURE 7:**
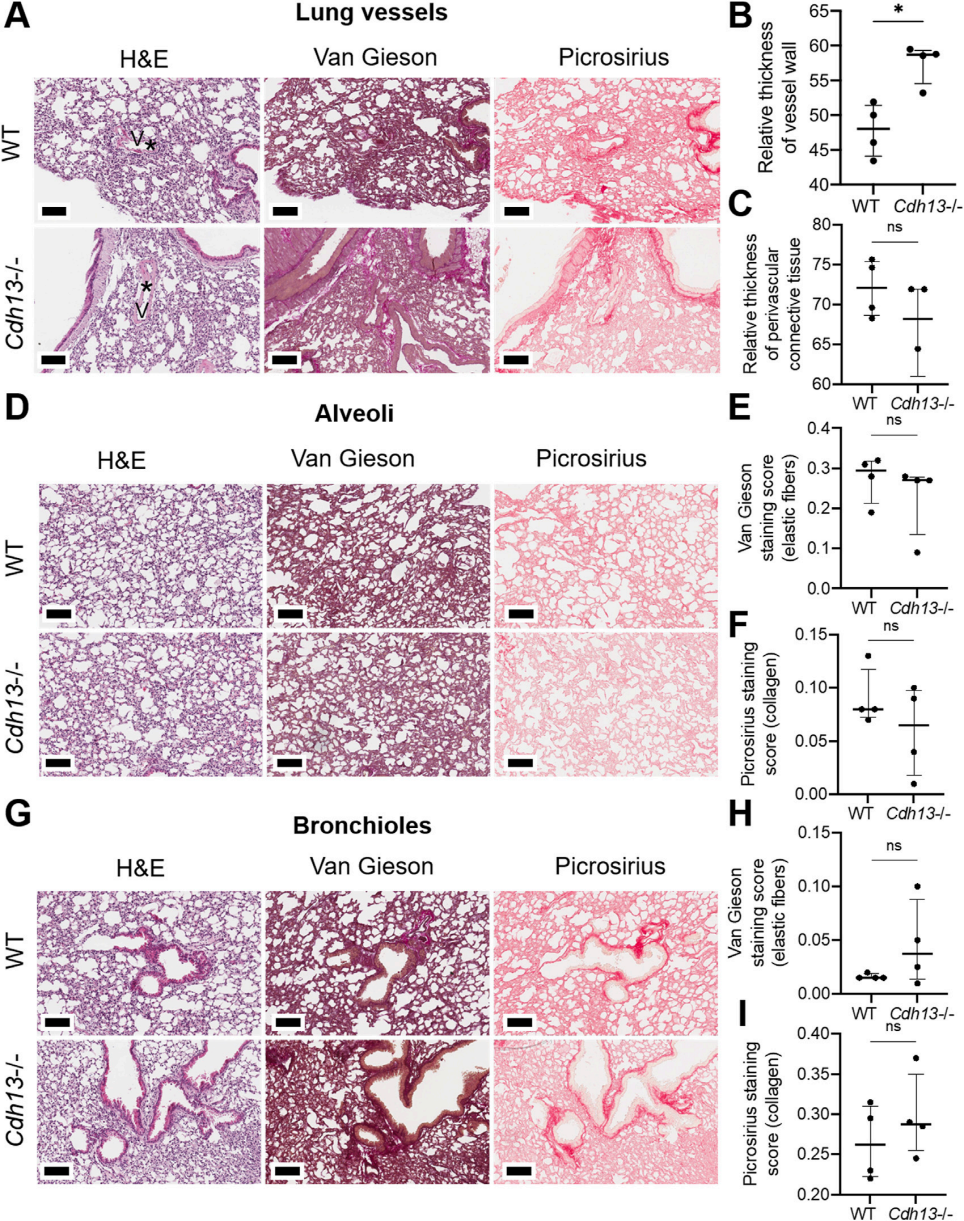
Histological assessment of fibrotic changes in the lung tissue of wild-type (WT) and T-cadherin knockout (*Cdh13* ^−/−^) mice on the 49th day after the angiotensin II administration. Tissue sections were stained with hematoxylin-eosin, Van Gieson, and picrosirius red. **(A)** Representative micrographs of histological staining of lung vessels. Asterisks point to the difference in vessel wall thickness. **(B)** Relative thickness of blood vessel walls presented as the percentage of vessel wall area (the difference between the total vessel area and luminal area) relative to the total vessel area. **(C)** Relative thickness of perivascular connective tissue presented as the percentage of connective tissue surrounding a blood vessel relative to the total area of the vessel and perivascular connective tissue. **(D)** Representative micrographs of histological staining of the alveoli. **(E)** The Van Gieson staining score in the alveoli presented as the ratio of positively stained area relative to the total area of the analyzed sample (staining score). **(F)** The picrosirius staining score in the alveoli presented as the ratio of positively stained area relative to the total area of the analyzed sample (staining score). **(G)** Representative micrographs of histological staining of the bronchioles. **(H)** The collagen area picrosirius staining score in the bronchioles presented as the ratio of positively stained area relative to the total area of the analyzed sample (staining score). **(I)** The collagen area picrosirius staining score in bronchioles presented as the ratio of positively stained area relative to the total area of the analyzed sample (staining score). Scale bar 100 μm. Data are presented as individual values, median and interquartile range, ns - non-significant, *p < 0.05, Mann-Whitney test.

We subsequently examined the influence of endothelial dysfunction on fibrosis development in kidneys and lungs. Following 10 weeks of angiotensin II administration, we isolated the lungs and kidneys from both WT and *Cdh13* ^−/−^ mice to conduct a comprehensive analysis. Morphometric analysis of renal tissue after angiotensin II treatment revealed a significant increase in vessel thickness in *Cdh13* ^−/−^ mice compared to control animals (*p* = 0.0286, Mann-Whitney, Figures 6A, B). However, there was no significant alteration in the thickness of perivascular connective tissue between the groups of mice (Figure 6C). Interestingly, no significant difference in the progression of fibrosis was observed in the renal cortex and perirenal adipose tissue between of *Cdh13* ^−/−^ mice and WT mice (Figures 6D–I). Similarly, an increase in vessel thickness was noted in *Cdh13* ^−/−^ mice compared to WT mice in the lung tissue (*p* = 0.0286, Mann-Whitney, Figures 7A, B), although there were no significant differences in perivascular connective tissue (Figure 7C). Additionally, no significant difference in the progression of fibrosis was observed in the alveoli and bronchioles between *Cdh13* ^−/−^ and WT mice (Figures 7D–I).

**FIGURE 8:**
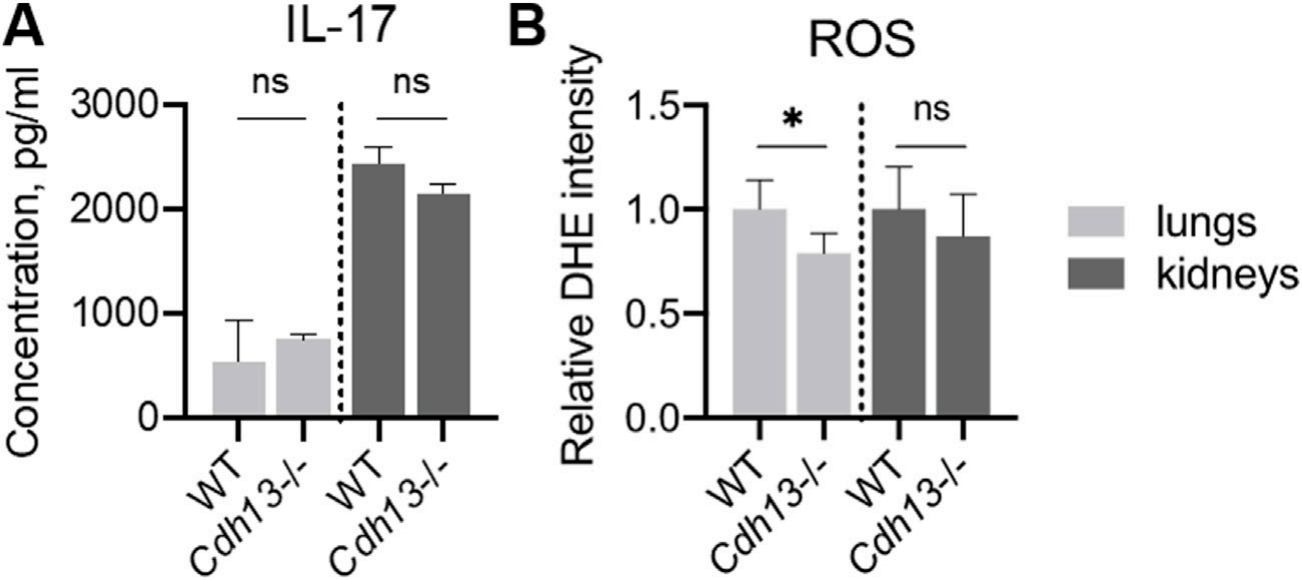
Inflammatory and oxidative stress markers in WT and T-cadherin knockout (*Cdh13* ^−/−^) mice in response to angiotensin II administration. **(A)** IL-17 concentrations in lung and kidney homogenates obtained from WT and *Cdh13* ^−/−^ mice after 10 weeks of angiotensin II administration (model of endothelial dysfunction). **(B)** Analysis of oxidized DHE content in the lungs and kidneys of WT and *Cdh13* ^−/−^ mice after 10 weeks of angiotensin II administration (model of endothelial dysfunction). Data are presented as median [interquartile range], ns - non-significant, *p < 0.05, Mann-Whitney test.

We next evaluated the concentration of interleukin-17 (IL-17), a pro-inflammatory cytokine, in lung and kidney homogenates using ELISA. No significant differences in IL-17 levels were observed between WT and *Cdh13* ^−/−^ mice in either tissue (Figure 8A). A significant decrease in reactive oxygen species (ROS) production was found in *Cdh13* ^−/−^ mice in lung extracts as assessed by quantitative DHE oxidation (*p* = 0.0286, Mann-Whitney test, Figure 8B). No significant differences in ROS production in kidney extracts were found in *Cdh13* ^−/−^ mice (Figure 8B).

**FIGURE 9:**
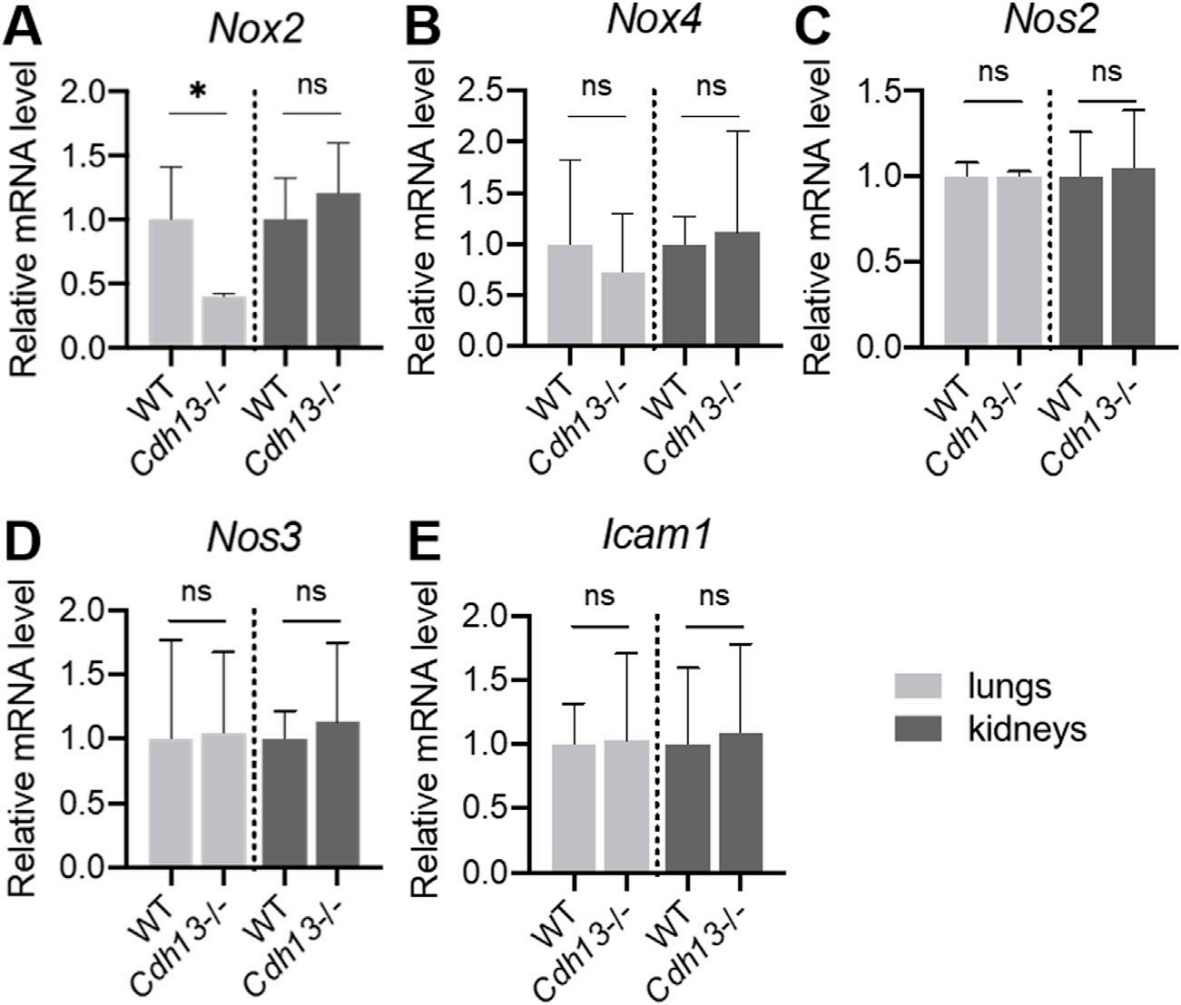
mRNA expression levels of *Nox2* **(A)**, *Nox4* **(B)**, *Nos2* **(C)**, *Nos3* **(D)** and *Icam1* **(E)** in homogenates of lungs and kidneys isolated from WT and *Cdh13* ^−/−^ mice after 10 weeks of angiotensin II administration (model of endothelial dysfunction). Data are presented as median [interquartile range], ns - non-significant, *p < 0.05, Mann-Whitney test.

Angiotensin II induces the transcription of four homologous forms of NADPH oxidases, *Nox2*, *Nox4*, *Nos2* (iNOS), and *Nos3* (eNOS), in the endothelium (Touyz et al., 2011). To investigate the impact of T-cadherin downregulation on NADPH oxidase expression, RT-qPCR was performed on lung and kidney tissues isolated from WT and *Cdh13* ^−/−^ mice after angiotensin II administration. The results revealed a significant downregulation (*p* = 0.0286, Mann-Whitney test, Figure 9A) of *Nox2* mRNA expression in the lungs of *Cdh13* ^−/−^ mice compared to WT. No significant differences were observed in the expression of *Nox4*, *Nos2* and *Nos3* isoforms between the two groups (Figures 9B–D). These findings suggest that T-cadherin downregulation selectively modulates *Nox2* expression in the lungs, potentially contributing to reduced ROS production (Figure 8B). In addition, we also examined the expression of *Icam1* which encodes endothelial cell adhesion molecule. No significant differences were observed in *Icam1* mRNA levels between WT and *Cdh13* ^−/−^ mice in either lung or kidney tissues (Figure 9E).

## 4 Discussion

It is widely recognized that SARS-CoV-2 predominantly affects the respiratory system, posing a significant risk of diminished lung function, including the development of pulmonary fibrosis as sequelae of COVID-19 (Zheng et al., 2023). Inflammation and endothelial dysfunction contribute to multi-organ injury in COVID-19 (Bonaventura et al., 2021; Ma et al., 2022). Molecular and cellular mechanisms related to COVID-19 pathophysiology still remain unclear, warranting further research to identify potential new targets.

In the present study we explored the role of T-cadherin in the pathogenesis of COVID-19 and underlying T-cadherin-related mechanism of pulmonary fibrosis and endothelial dysfunction. We utilized experimental *in vitro* and *in vivo* models, along with human post-mortem tissue section analysis, to comprehensively examine these phenomena. Engaged in a variety of intracellular communication processes, T-cadherin plays a multifaceted role, spanning from regulating cell-cell adhesion and endothelial permeability through homophilic interactions to acting as a receptor for two ligands, HMW adiponectin and LDL, possibly affecting inflammation and systemic metabolism (Tkachuk et al., 1998; Hug et al., 2004; Rubina et al., 2021). While adiponectin exerts multiple protective effects, elevated LDL levels are associated with a risk of atherogenesis and compromised vessel function. Consequently, T-cadherin emerges as a pivotal molecular hub, yet the link between *CDH13* gene and COVID-19 symptoms has not been investigated so far.

In humans, systemic adiponectin level positively correlates with proper lung function in healthy adults. Reduced circulating adiponectin levels have been associated with severe subclinical lung inflammation, fibrosis, and diminished lung function (Kim et al., 2020). In pulmonary diseases such as asthma and COPD (chronic obstructive pulmonary disease), the precise role of adiponectin remains to be determined (Garcia and Sood, 2012; Salvator et al., 2021).

Our initial findings from the immunohistochemistry analysis of lung tissues obtained from COVID-19 patients revealed a notable decrease in T-cadherin expression, particularly evident in the stromal regions, with no significant differences detected in bronchioles or blood vessels (Figure 1). The pertinent literature data indicates that a decrease in T-cadherin levels leads to a diminished sequestration of adiponectin by responsive tissues, such as muscles, adipose tissue, heart and blood vessels [cited in (Barbalho et al., 2023)]. Consequently, the downregulation of adiponectin-activated signaling pathways can deplete the protective effects of adiponectin, such as enhanced fatty acid oxidation and glucose uptake, reduced apoptosis, augmented vasodilation, and decreased inflammation and fibrosis in these tissues (Reiterer et al., 2021; Barbalho et al., 2023). The existing data on adiponectin as a diagnostic or prognostic marker in COVID-19 has been inconsistent, often limited by small cohort sizes or confounding factors unrelated to COVID-19, such as BMI (Grewal and Buechler, 2023). Moreover, serum adiponectin levels can vary among COVID-19 patients, and there is evidence that hyperadiponectinemia directly correlates with the emergence of a SARS-CoV-2 inflammation-induced “adiponectin paradox”. In other words, despite the elevated serum levels of adiponectin, which hypothetically may trigger its protective effects, adiponectin sequestration in tissues and subsequent signal transduction through its receptors are impaired, particularly among individuals with obesity (Barbalho et al., 2023). Therefore, we hypothesized that T-cadherin may influence metabolic regulation and disease progression in COVID-19. In addition, the interplay between polymorphisms (SNPs) in the *CDH13* gene, which encodes T-cadherin, and plasma adiponectin levels suggests the existence of a feedback regulatory loop, further supporting this hypothesis (Jee et al., 2010; Wu et al., 2010; Rubina et al., 2021). While the current study identified significant downregulation of T-cadherin expression, aligning with recent findings on decreased plasma levels of T-cadherin (Rosario-Rodríguez et al., 2024), further investigation into the role of T-cadherin is necessary to elucidate its involvement in COVID-19 pathogenesis.

Our immunohistochemistry analysis of lung tissues of COVID-19 patients revealed heightened levels of VE-cadherin in both lung stroma and blood vessels along with a reduced expression of β-catenin in COVID-19 patients compared with healthy controls (Figure 2). We have previously demonstrated that overexpression of T-cadherin in cultured endothelial cells (HUVECs) triggers clathrin-dependent endocytosis of VE-cadherin with its subsequent degradation in lysosomes, resulting in the disruption of endothelial barrier function and increased permeability (Semina et al., 2014). Consistent with these data, our results on human endothelial Ea.hy926 cells demonstrated a decrease in mRNA expression levels of both VE-cadherin and β-catenin upon T-cadherin overexpression (Figure 3). Our data on the elevated VE-cadherin expression (Figures 2, 3) in lung samples of COVID-19 patients are in line with the reduced T-cadherin expression (Figure 1), though in contrast with the recently published results on reduced VE-Cadherin expression during SARS-CoV-2 viral infection (Nader and Kerrigan, 2022; Xu et al., 2023). It has been suggested that in COVID-19 patients, VE-cadherin undergoes downregulation or internalization, causing a change of its localization in cell-cell contacts, thereby contributing to the loss of endothelial barrier function. Soluble VE-cadherin (sVE-cadherin) was listed among the endothelial dysfunction markers related to endothelial inflammation, thrombosis, glycocalyx damage, vascular tone in COVID-19 patients (Xu et al., 2023). Our results do not unambiguously clarify the functionality of VE-cadherin, particularly given the heightened VE-cadherin staining in the stroma of COVID-19 patients (Figure 2), and may indicate VE-cadherin potential cleavage or intracellular localization. Reduced β-catenin expression along with elevated VE-cadherin (Figure 2) may point to the overall dysregulated endothelial cell-cell contact organization and function typical of SARS-CoV-2 viral infection (Xu et al., 2023; 2024). The increased expression of VCAM-1 in small vessels, and in both the intima and media of large vessels (Supplementary Figure S3), is in line with the known elevated expression of this marker upon inflammation, including COVID-19 infection (Xu et al., 2023).

To discern the potential role of T-cadherin in the development of fibrosis or endothelial dysfunction, we enrolled two animal models involving wild-type (WT) and T-cadherin knockout (*Cdh13* ^−/−^) mice: a bleomycin-induced model of lung injury and an angiotensin II-induced model of endothelial dysfunction.

*Cdh13* ^−/−^ mice displayed considerably diminished lung fibrosis compared to the controls (Figure 4B). A plausible explanation for this phenomenon can be that in bleomycin-induced model of lung fibrosis, the T-cadherin deficiency, leading to an increase in plasma adiponectin concentration, ensures adiponectin protective effects in the lungs. This hypothesis finds support in the study by Williams et al., where the authors challenged control and knockout mice (T-cadherin knockout, adiponectin knockout, T-cadherin and adiponectin double knockouts) with aerosolized ovalbumin (OVA) and performed subsequent histological analysis of the lungs. Their findings revealed reduced inflammation (lymphocytes in the airspaces and secreted IL-17, IL-13) around the airways, and diminished mucous cell hyperplasia due to upregulated plasma adiponectin (Denzel et al., 2010). This is consistent with the findings by Denzel et al., and results from our lab, which demonstrated that T-cadherin deficiency leads to elevated plasma adiponectin levels in T-cadherin knockout mice (Denzel et al., 2010; Popov et al., 2023).

The second potential explanation for these results may lie in T-cadherin’s ability to downregulate the secretion of surfactant protein D (SP-D), as previously demonstrated in human lung A549 cells (a human bronchioloalveolar carcinoma cell line with properties of type-II alveolar cells) (Takeuchi et al., 2001). SP-D performs an immunomodulatory function, inhibiting T-lymphocyte proliferation and interleukin (IL)-2 production (Takeuchi et al., 2001). Elevated serum SP-D levels have been proposed as a biomarker for the severity of COVID-19, since patients with severe COVID-19 pneumonia exhibited increased plasma levels of SP-D gradually decreasing in the course of recovery period (Tong et al., 2021). Therefore, T-cadherin’s capacity to decrease SP-D production may mitigate the inflammatory response through an undisclosed mechanism, rather than through adiponectin binding to T-cadherin. This might underpin the protective effect of T-cadherin deficiency in lungs.

Angiotensin II, a key component of the renin–angiotensin–aldosterone system (RAAS), is known to play an important role in the pathophysiology of cardiovascular and renal diseases (Weiss et al., 2006; Dong et al., 2019). In the present study, we employed a murine model of chronic exogenous angiotensin II administration via daily intraperitoneal injection, which has been previously established for investigating endothelial dysfunction pathophysiology (Trejo-Moreno et al., 2021). This model of angiotensin II administration was shown to induce a complete endothelial dysfunction, including hypertension, vascular remodeling, prooxidant and proinflammatory activity (resulting in increased production of TNFα, IL-1β, IL-17A, IL-4, TGFβ, and IL-10 in the kidney, and systemic soluble VCAM, ROS, and ICAM-1 expression), and organ injury (Trejo-Moreno et al., 2021).

In both WT and *Cdh13* ^−/−^ mice we observed significant alterations in systolic pressure, indicating vascular wall remodeling, a crucial aspect of endothelial dysfunction that impedes blood flow and promotes hypertension. However, no significant difference was detected between the animal groups (Figure 5). Similarly, no differences were found either in diastolic pressure or heart rate between the groups (Figure 5). Of note, our previous study showed that resting blood pressure in *Cdh13* ^−/−^ mice was marginally higher than in the controls, albeit not statistically significant (Popov et al., 2023). Therefore, T-cadherin deficiency under the conditions of angiotensin II-induced endothelial dysfunction did not exert any major effects.

Among the parameters examined, a significant increase was observed in vessel thickness in renal tissue and lungs of *Cdh13* ^−/−^ mice compared to control animals (Figures 6, 7). However, no significant difference in the thickness of perivascular connective tissue was noted in either renal tissue (Figure 6) or lungs (Figure 7) between the groups. No significant differences were observed in *Icam1* mRNA levels between WT and *Cdh13* ^−/−^ mice in either lung or kidney tissues (Figure 9E). No differences in the progression of fibrosis were observed between the groups in the renal cortex and perirenal adipose tissue, as well as in the alveoli and bronchioles (Figures 6, 7). Additionally, no significant differences in IL-17 levels, as assessed by ELISA, were observed in lung and kidney homogenates between WT and *Cdh13* ^−/−^ mice in either tissue (Figure 8A). Therefore, in comparison to the controls, *Cdh13* ^−/−^ mice exhibited only mild signs of vascular damage due to angiotensin II-induced endothelial dysfunction.

Despite the described effects of T-cadherin deficiency on vessel thickness pointing to vascular injury, we revealed a significant decrease in ROS production and *Nox2* mRNA expression level in the lungs of *Cdh13* ^−/−^ mice compared to WT. Oxidative stress plays an important role in the pathophysiology of endothelial dysfunction and the related cardiovascular diseases, such as hypertension, atherosclerosis, diabetes, cardiac hypertrophy, heart failure, and ischemia–reperfusion (Massaeli and Pierce, 1995; Cave et al., 2006). A family of NADPH oxidases are crucial for redox signaling (Cave et al., 2006). The catalytic component of the NADPH oxidase complex in humans comprises six homologous forms of NOX, which share the capacity to generate superoxide and other downstream ROS. NOX2 and NOX4 are expressed in the endothelium, and angiotensin II can induce their transcription (Trejo-Moreno et al., 2021). We assessed ROS production by quantitative DHE oxidation assay and noted a significant decrease in ROS levels in lung extracts from *Cdh13* ^−/−^ mice (Figure 8B). Consistent with this, the RT-qPCR analysis revealed a notable decrease in *Nox2* mRNA expression (but not in *Nox4*, *Nos2*, or *Nos3*) in the lungs of *Cdh13* ^−/−^ mice compared to the controls (Figure 9A).

Here, we provide a comprehensive analysis unveiling several fundamental features of T-cadherin expression and function. First, we revealed a significant decrease in T-cadherin expression in post-mortem lung samples from COVID-19 patients. This downregulated expression correlated with elevated level of VE-cadherin and reduced level of β-catenin, suggesting a disruption in endothelial cell-cell contact integrity and function. This inverse correlation between T-cadherin and VE-cadherin expression was further validated using human endothelial Ea.hy926 cells *in vitro*: overexpression of T-cadherin resulted in a decrease in VE-cadherin mRNA expression. Furthermore, through experiments involving *Cdh13* ^−/−^ mice, we demonstrated that T-cadherin deficiency confers protection against bleomycin-induced lung fibrosis. The elevated levels of ROS and NADPH oxidases serve as markers of endothelial dysfunction, culminating in the impaired vascular endothelial integrity. We found that T-cadherin deficiency offers protection against excessive ROS production and upregulation of Nox2 mRNA expression induced by angiotensin II treatment. Further detailed studies on the underlying mechanisms involving T-cadherin are needed to dissect its specific role in pathogenesis of COVID-19, endothelial dysfunction and lung fibrosis.

## Data Availability

The original contributions presented in the study are included in the article/Supplementary Material, further inquiries can be directed to the corresponding author.
