# Supplementary material for: New evidence for T-cadherin in COVID-19 pathogenesis, endothelial dysfunction, and lung fibrosis
Source: Front Cell Dev Biol. 2025 Mar 5;13:1476329. doi: 10.3389/fcell.2025.1476329 (PMC11920143; doi:10.3389/fcell.2025.1476329)
Supplement: Supplementary file 1 [file DataSheet1.docx]

Supplementary Material

# Materials and Methods.

**Double immunofluorescence staining**

Human lung tissues were fixed in formalin (Sintacon, Russia), embedded in paraffin (BioVitrum, Russia), and sectioned into 5 μm slices, which were mounted on glass slides. Deparaffinization was performed with xylene (BioVitrum, Russia), followed by rehydration in graded methanol solutions (100%, 95%, and 70%) (Ecolan, Russia). To prevent non-specific binding, sections were treated with 10% normal donkey serum (Sigma-Aldrich, USA), washed in PBS, and subjected to double immunostaining with the following primary antibody combinations: T-cadherin (Affinity Biosciences, AF5203, USA, 1:100) and anti-CD31 (Dako, clone JC70A, USA, 1:100); T-cadherin and E-cadherin (Santa Cruz, sc-8426, USA, 1:100); T-cadherin and α-SMA (Dako, clone 1A4, USA, 1:100). After washing three times with PBS, the samples were incubated with secondary antibodies conjugated to Alexa Fluor®594 and Alexa Fluor®488 (1:1000, Molecular Probes). Nuclei were counterstained with DAPI (Sigma-Aldrich, USA, 1:10,000). The samples were mounted using Aqua Poly Mount medium (Polysciences, USA). Images were captured using a Zeiss LSM 780 confocal microscope with ZEN 2010 software with the same confocal gain and offset settings. DAPI, Alexa Fluor®488, and Alexa Fluor®594 were sequentially excited using lasers with wavelengths of 405, 488, and 594 nm, respectively.

# Results.

# Supplementary Figures


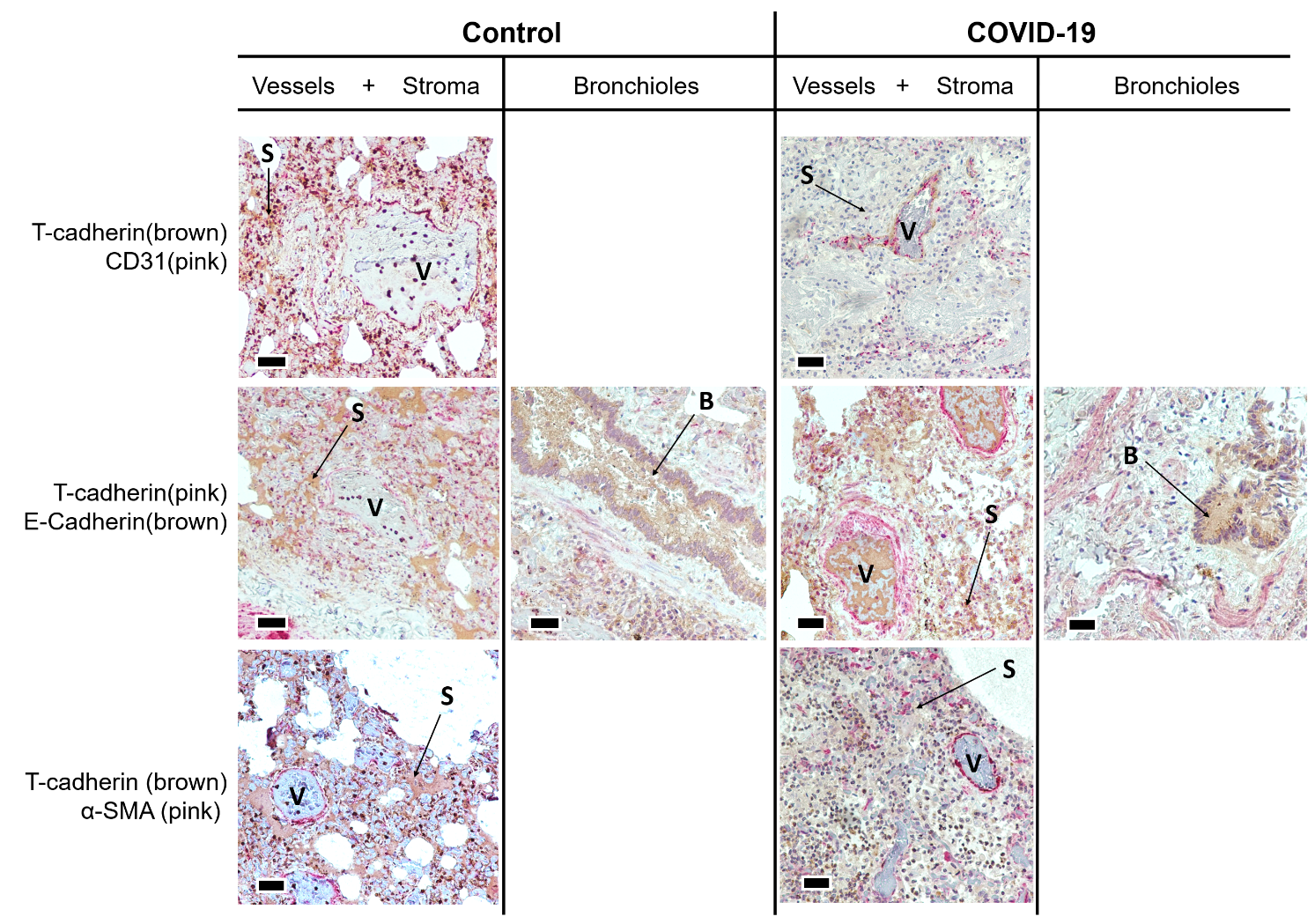


**Supplementary figure 1**. Histological assessment of T-cadherin, CD31, a-SMA and E-cadherin expression detected in lung tissue of healthy controls (Control) and COVID-19 patients. Representative section of lung tissues double immunostained for T-cadherin (brown or pink), for CD31 (pink) to reveal blood vessels, for E-cadherin (brown) in bronchioles and for α-SMA (pink) to reveal the stroma, counterstained with hematoxylin; S – stroma, V – vessels, B – bronchioles. Scale bar 100 μm.


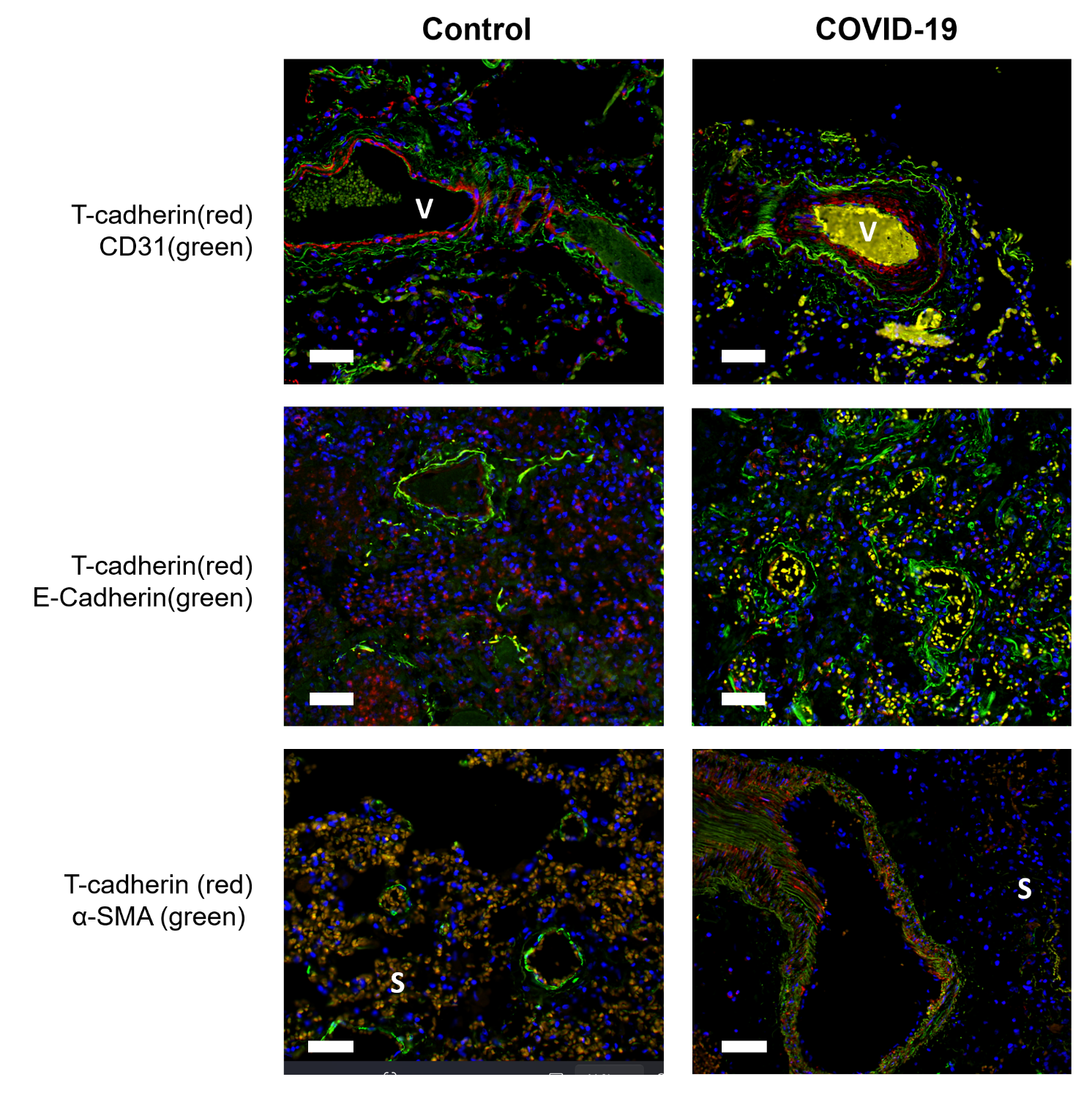


**Supplementary figure 2**. Immunofluorescent staining for T-cadherin, CD31, α-SMA and E-cadherin expression using lung tissue of healthy controls (Control) and COVID-19 patients. Representative section of lung tissues double immunofluorescence stained for T-cadherin (red), vessels for CD31 (green), bronchioles for E-cadherin (green) and stroma for α-SMA (green) counterstained with DAPI (blue). S – stroma, V – vessels. Scale bar 100 μm.


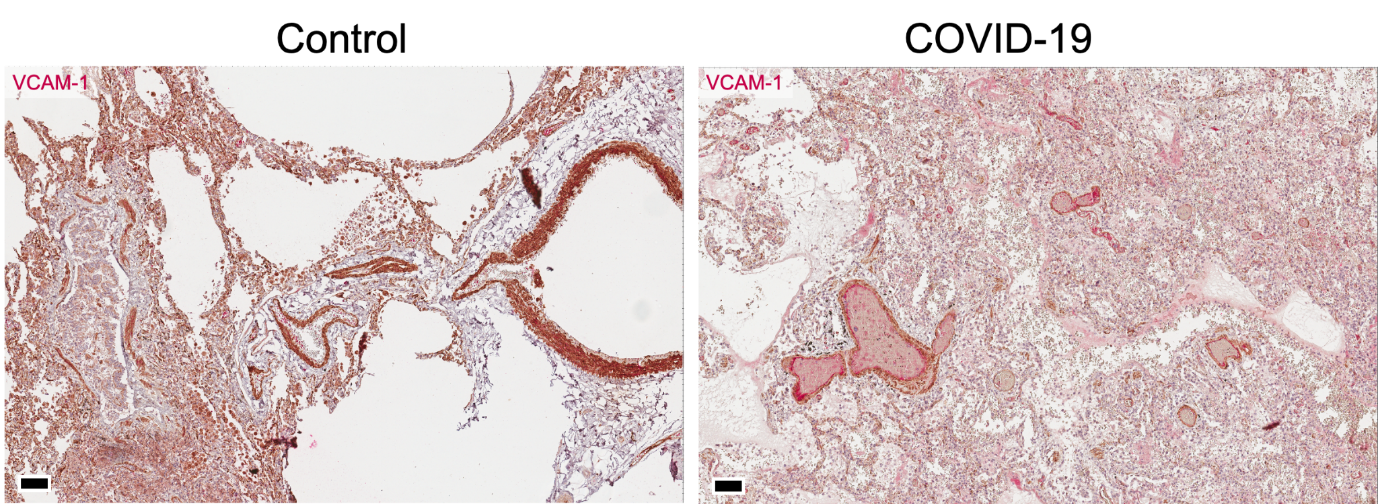


**Supplementary figure 3**. VCAM-1 expression detected in lung tissue of healthy controls and COVID-19 patients. Representative sections of parenchymal lung tissue from healthy control and COVID-19 patients stained for VCAM-1 and revealed with ImmPACT Vector Red Substrate (pink) and counterstained with hematoxylin. Scale bar 100 μm.
